# Detection of bacterial endosymbionts in freshwater crustaceans: the applicability of non-degenerate primers to amplify the bacterial 16S rRNA gene

**DOI:** 10.7717/peerj.6039

**Published:** 2018-12-14

**Authors:** Monika Mioduchowska, Michał Jan Czyż, Bartłomiej Gołdyn, Adrianna Kilikowska, Tadeusz Namiotko, Tom Pinceel, Małgorzata Łaciak, Jerzy Sell

**Affiliations:** 1 Department of Genetics and Biosystematics, Faculty of Biology, University of Gdansk, Gdansk, Poland; 2 Research Centre of Quarantine, Invasive and Genetically Modified Organisms, Institute of Plant Protection—National Research Institute, Poznan, Poland; 3 Department of General Zoology, Institute of Environmental Biology, Faculty of Biology, Adam Mickiewicz University, Poznan, Poland; 4 Animal Ecology, Global Change and Sustainable Development, KU Leuven, Leuven, Belgium; 5 Centre for Environmental Management, University of the Free State, Bloemfontein, South Africa; 6 Polish Academy of Sciences, Institute of Nature Conservation, Krakow, Poland

**Keywords:** Microbiome, Fairy shrimp, Anostraca, Cladocera, Ostracoda, Temporary ponds

## Abstract

Bacterial endosymbionts of aquatic invertebrates remain poorly studied. This is at least partly due to a lack of suitable techniques and primers for their identification. We designed a pair of non-degenerate primers which enabled us to amplify a fragment of ca. 500 bp of the 16S rRNA gene from various known bacterial endosymbiont species. By using this approach, we identified four bacterial endosymbionts, two endoparasites and one uncultured bacterium in seven, taxonomically diverse, freshwater crustacean hosts from temporary waters across a wide geographical area. The overall efficiency of our new WOLBSL and WOLBSR primers for amplification of the bacterial 16S rRNA gene was 100%. However, if different bacterial species from one sample were amplified simultaneously, sequences were illegible, despite a good quality of PCR products. Therefore, we suggest using our primers at the first stage of bacterial endosymbiont identification. Subsequently, genus specific primers are recommended. Overall, in the era of next-generation sequencing our method can be used as a first simple and low-cost approach to identify potential microbial symbionts associated with freshwater crustaceans using simple Sanger sequencing. The potential to detected bacterial symbionts in various invertebrate hosts in such a way will facilitate studies on host-symbiont interactions and coevolution.

## Introduction

Many invertebrate species have endosymbiotic bacteria that exert various effects on the biology of their host. The benefits derived from both obligatory and facultative dependence include: mutual effects on defense, nutrition, development and reproduction (e.g., Pontes & Dale, 2006; Pais et al., 2008). The best studied examples of such interactions include cases of *Wolbachia* (Hurst et al., 2000) and *Cardinium* (Gotoh, Noda & Ito, 2007). These bacteria can interfere with host reproduction by inducing cytoplasmic incompatibility and shifts in sexual selection (Zhang, Zhao & Hong, 2012). Although such interactions were traditionally regarded as harmful to the host, an increasing body of evidence also supports host fitness benefits associated with the presence of symbionts (Zug & Hammerstein, 2018).

Most information on obligatory host-bacteria associations comes from terrestrial invertebrates and insects in particular (Chaston & Goodrich-Blair, 2010). However, associations with symbiotic bacteria have also been observed in aquatic invertebrates (both marine and freshwater), affecting both ecology and evolution of the hosts (Ott, Bright & Bulgheresi, 2004; Dubilier, Bergin & Lott, 2008; Dattagupta et al., 2009). Some endosymbionts, such as *Rickettsia* species of the *torix* group, are widespread in freshwater metazoans including larvae of biting midges, but are much more rare in terrestrial species (Pilgrim et al., 2017). Furthermore, heritable microbes (inherited through the egg cytoplasm during the reproduction of their hosts) have also been recently reported in freshwater snails, hydrozoans, leeches and arthropods (e.g., Perkins, Budinoff & Siddall, 2005).

The study of obligatory bacterial endosymbionts is complicated by the fact that they cannot be cultured in the laboratory and that only molecular techniques are useful in their detection. Also, only DNA of bacteria that make up at least 1% of the host bacterial community can typically be amplified with broad-range universal primers (Muyzer et al., 1996; Yamamoto, 2002; Wang et al., 2016). More sensitive genus- or species-specific primers are required to overcome these problems (Gottlieb et al., 2006).

Temporary ponds are typically shallow aquatic ecosystems that dry out at unpredictable moments due to changing weather conditions. Despite drying, they are often home to diverse aquatic communities of species that have evolved ways to survive the dry periods (Williams, 2006). Host-endosymbiont relationships in freshwater invertebrates from such systems, as well as their geographical and ecological distribution have hardly been studied (Toft & Andersson, 2010). These fragile habitats are undergoing a dramatic decline worldwide since they are prone to land alterations and destructions by human activity and climatic changes (De Meester et al., 2005).

Invertebrates from temporary ponds are generally characterized by rapid growth and maturation, short-life spans, small sizes, and high reproductive output. All large branchiopod crustaceans (clam, fairy and tadpole shrimps) and many cladocerans, copepods and ostracods are temporary pond specialists. They all have the ability to produce resting (dormant or diapausing) eggs (cysts) that can withstand extended droughts in a state of dormancy (Smith, 2001; Williams, 2006).

So far there are no data showing that bacterial endosymbionts of freshwater or marine animals manipulate their host reproduction mode. However, it seems probable that inhabiting temporary ponds could be facilitated in many species by the asexual reproduction (e.g., ameiotic parthenogenesis) or a modification of the sexual reproduction mode (e.g., androdioecy as well as masculinization, feminization) associated with bacterial endosymbionts such as *Wolbachia* (Kageyama, Narita & Watanabe, 2012). With this background, we attempted to identify *Wolbachia* (the most prevalent bacterial endosymbiont of invertebrates) and other related bacteria in taxonomically diverse crustacean species from astatic freshwater ponds using newly designed non-degenerate primers to amplify the bacterial 16S rRNA gene fragment. However, due to the fact that the term “symbiont” in the comprehensive meaning concerns both endosymbiont as well as pathogenic bacteria (since the host-symbiont interactions refer to mutualism, parasitism and commensalism) we opted for a more specific nomenclature to describe identified bacterial species and to define these as “endosymbionts” (Perez-Brocal, Latorre & Moya, 2011).

## Materials and Methods

Preliminary detection and identification of bacterial endosymbionts of the genus *Wolbachia* in the anostracan *Branchipus schaefferi* was performed using 16S rRNA sequence primers (WF 5′–CGGGGGAAAATTTATTGCT-3′ and WR 5′-AGCTGTAATACAGAAAGGAAA-3′) and PCR protocols by Singh et al. (2013). Since the amplification success was limited, we designed new, non-degenerate primers *in silico*. Using the existing WF and WR primers, we obtained a 544 bp-long 16S rRNA sequence of *Wolbachia* that was deposited in GenBank under accession number MH454491. This sequence was further used to design internal primers to amplify a 458 bp fragment with the Primer3 v. 0.4.0 (Untergasser et al., 2012) software package. The sequence of the newly developed forward WOLBSL primer was 5′-GCTAGTTGGTGGAGTAATAGCC-3′, and that of the reverse WOLBSR was 5′-GACTACCAGGGTATCTAATCCTG-3′.

**Figure 1:**
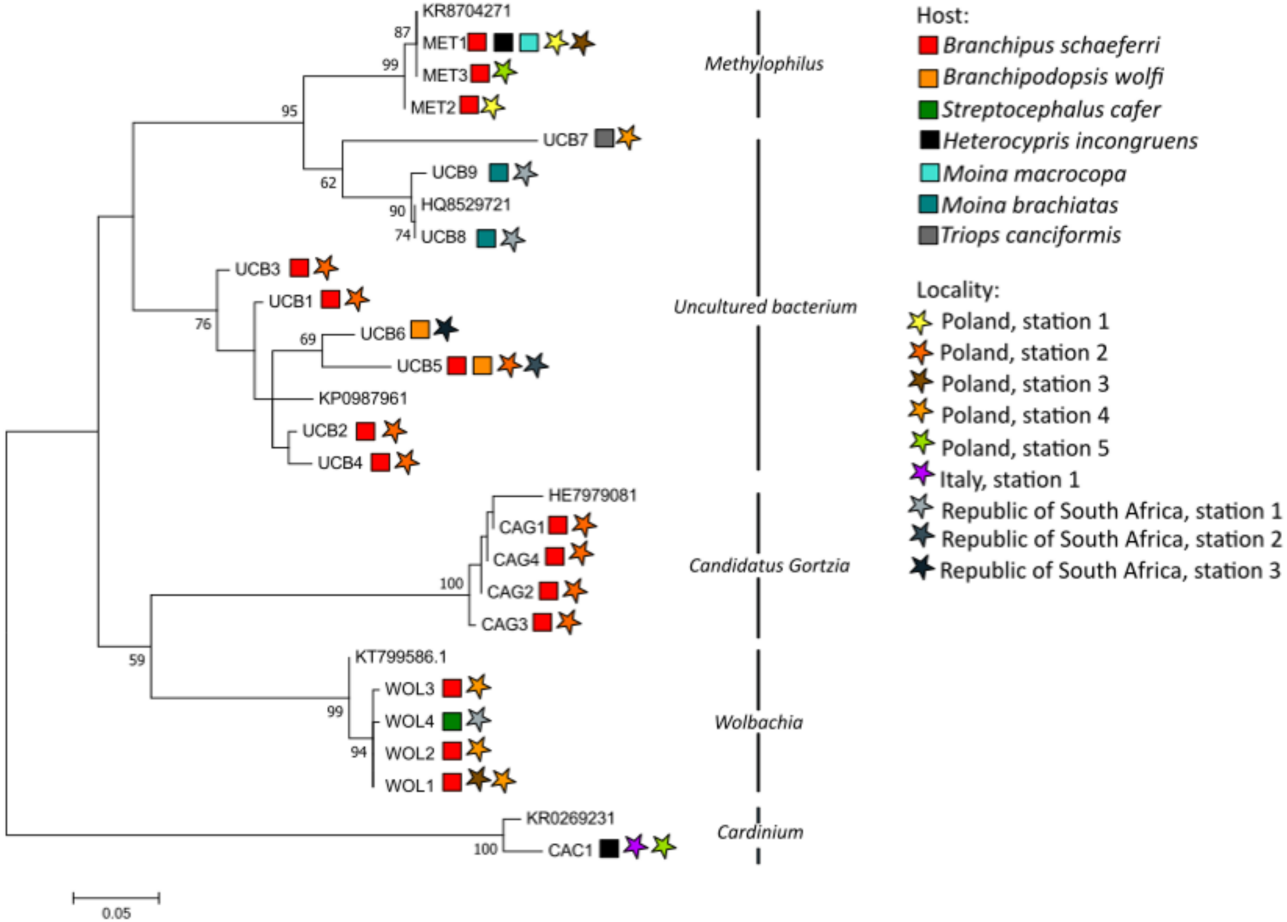
Maximum likelihood tree showing the relationships among bacterial sequences obtained in our study from freshwater crustacean hosts and closely matching query sequences downloaded from GenBank.

The designed WOLBSL and WOLBSR primers were subsequently used for detection of *Wolbachia* in other potential host species. We tested them on available crustacean material collected from nine distant astatic ponds, i.e., five from various regions across Poland, one from Italy and three from South Africa. Crustaceans were identified to the species level based on morphological criteria. Data on the sampling sites and the species selected to test the general applicability of the designed primers are listed in Table 1 and Fig. 1.

**Table 1:** Identified bacterial sequences in freshwater crustacean hosts and locality of sampling sites.

**Freshwater crustacean hosts**	**Locality of astatic ponds:** **country; station code; coordinates/** **number of sampled host individuals**	**Identified bacterial sequences: haplotype code (GenBank accession number of sequences, sequence length)**
***Branchipus schaefferi***	Poland; Station 1; 54°26′N, 17°03′E/5	•***Methylophilus***: MET1 (MH447385, 483 bp; MH447386, 514 bp; MH447387, 524 bp), MET2 (MH447389, 497 bp)
	Poland; Station 2; 53°32′N, 15°48′E/14	•***Candidatus*Gortzia:** CAG1 (MH450232, 452 bp; MH450233, 452 bp), CAG2 (MH450235, 452 bp), CAG3 (MH450236, 452 bp), CAG4 (MH450234, 452 bp) •**Uncultured bacterium:** UCB1 (MH447689, 394 bp), UCB2 (MH447690, 394 bp), UCB3 (MH447691, 392 bp), UCB4 (MH447692, 392 bp), UCB5 (MH447693, 432 bp; MH447694, 433 bp)
	Poland; Station 3; 53°08′N, 16°48′E/5	•***Wolbachia***: WOL1 (MH447361, 458 bp; MH447362, 458 bp) •***Methylophilus***: MET1 (MH447388, 503 bp)^a^
	Poland; Station 4; 52°29′N, 16°51′E/5	•***Wolbachia***: WOL1 (MH447363, 458 bp), WOL2 (MH447364, 458 bp ), WOL3 MH447365, 458 bp) •***Spirobacillus***: SPR1 (MH450240, 471 bp) •***Undibacterium***: UND1 (MH450134, 297 bp)
	Poland; Station 5; 49°53′N, 20°11′E/2	•***Methylophilus***: MET3 (MH447390, 509 bp)
***Branchipodopsis wolfi***	South Africa; Station 2; 28°51′S, 27°13′E/5	•**Uncultured bacterium:** UCB5 (MH447695, 487 bp; MH447696, 487 bp; MH447697, 495 bp)
	South Africa; Station 3; 28°51′S, 27°13′E/2	•**Uncultured bacterium:** UCB6 (MH447698, 467 bp)
***Streptocephalus cafer***	South Africa ; Station 1; 28°47′S, 21°00′E/1	•***Wolbachia***: WOL4 (MH447357; 458 bp)
***Heterocypris incongruens***	Italy; Station 1; 41°16′N, 14°10′E/2	•***Cardinium***: CAC1 (MH376282, 531 bp; MH376281, 500 bp)
	Poland; Station 5; 49°53′N, 20°11′E/2	•***Cardinium***: CAC1 (MH376280, 500 bp)
	Poland; Station 3; 53°08′N, 16°48′E/2	•***Methylophilus***: MET1 (MH447391, 523 bp )
***Moina macrocopa***	Poland; Station 3; 53°08′N, 16°48′E/1	•***Methylophilus***: MET1 (MH447392, 518 bp )
***Moina brachiata***	South Africa; Station 1; 28°47′S, 21°00′E/3	•**Uncultured bacterium:** UCB8 (MH447700, 491 bp; MH447701, 491 bp, UCB9 MH447702, 491 bp)
***Triops cancriformis***	Poland; Station 4; 52°29′N, 16°51′E/1	•**Uncultured bacterium:** UCB7 (MH447699, 372 bp)

**Notes.**

a Isolate from cyst.

The total DNA from seven crustacean species (approximately 3 mm^3^ of thorax tissues of *Branchipus schaefferi*, *Branchipodopsis wolfi*, *Streptocephalus cafer*, *Triops cancriformis*; cysts of *Branchipus schaefferi*; whole specimens of *Heterocypris incongruens*, *Moina brachiata* and *Moina macrocopa*) was isolated according to the Biotrace Genomic Extraction GPB Mini Kit (GenoPlast) protocol. Extreme care was taken to avoid bacterial contamination in our experiment—crustacean individuals were surface-cleaned by washing with sterile water and all steps were carried out in a sterilized laminar flow hood. PCR reactions were performed in 20 µL volume containing 0.8x JumpStart Taq ReadyMix (1 U of JumpStart Taq DNA polymerase, 4 mM Tris-HCl, 20 mM KCl, 0.6 mM MgCl_2_, 0.08 mM of dNTP; SigmaAldrich, Germany), 0.4 µM of both newly designed primers and about 100 ng of DNA. The 16S rRNA gene fragment was amplified under the following conditions: initial denaturation at 94 °C for 5 min followed by 44 cycles of 94 °C for 30 s, 51.2 °C for 40 s and 72 °C for 2 min and ending with 72 °C for 5 min. Amplified products were separated by 1% agarose gel electrophoresis in a 1×SB buffer and visualized with ethidium bromide under UV light. All PCR products were purified by alkaline phosphatase and exonuclease I (Thermo Scienific, USA) treatment (incubation at 37 °C for 60 min and heating at 65 °C for 15 min) and sequenced following the BigDye™ terminator cycle sequencing method.

The comparison with GenBank records and the homology search was carried out with Blast application using blastn searches (Altshul et al., 1990). We accepted only the results which indicated high identity ≥ 95% with a query cover near 100% and an *E* value near 0.0.

Sequences were quality checked and trimmed to the same length in BioEdit (Hall, 1999) and a consensus sequence was created for each individual. The sequences were aligned using Clustal ×1.81 (Thompson et al., 1997) under default settings. Although the 16S rRNA alignment contained several indels, all sequences were readily readable and could be unambiguously aligned. Haplotypes were retrieved using DnaSP v.5.10.01 software (Librado & Rozas, 2009). All obtained sequences were deposited in GenBank under accession numbers given in Table 1.

To infer the phylogenetic relationships among the bacterial sequences obtained in our study, we calculated maximum-likelihood trees in MEGA7 (Kumar, Stecher & Tamura, 2016). Closely matching reference sequences from GenBank were included to verify the identity of the new sequences (Accession numbers are given in Table 1). The most appropriate evolutionary model was determined with jModelTest 2 (Darriba et al., 2012). Based on the Akaike Information Criterion (AIC) and Bayesian Inference Criterion (BIC), the HKY +I +G model of evolution was chosen. For the assessment of the node support 1,000 bootstrap iterations were run.

In order to verify prevalence of the bacterial species identified in our study, bacterial sequence records were downloaded from the NCBI Nucleotide database using R 3.4.4 (R Core Team, 2018) and the package rentrez 1.2.1 (Winter, 2017). The query was constructed as “<genus>[ORGN]” where for the genus we applied: *Cardinium*, Gortzia, *Methylophilus*, *Spirobacillus*, *Undibacterium* and *Wolbachia*. For uncultured bacteria we used two queries: “uncultured bacterium[ORGN]” to check the overall number of sequences in the NCBI database, and “uncultured bacterium[ORGN] AND endosymbiont[WORD]” to limit the number of sequence records to those which were reported as endosymbionts.

## Results

In our experiment, we used a total of 50 isolates from seven freshwater invertebrate species inhabiting temporary waters in Europe and South Africa. The overall efficiency of designed primers to amplify bacterial 16S rRNA gene was 100%. We obtained 38 bacterial good quality 16S rRNA sequences of a length of ca. 500 bp (Table 1). Poor sequencing results, despite good PCR products for seven isolates of *B. schaefferi*, three isolates of *B. wolfi* and two isolates of *H. incongruens*, were most likely due to genome mixing among several bacterial species. This was anticipated since our primers can identify various bacterial species.

The obtained bacterial 16S rRNA gene sequences (ca. 500 bp) closely matched bacterial endosymbiont sequences deposited in GenBank. We constructed a phylogenetic tree with both: all the existing closely matching query sequences in GenBank and the newly generated sequences to confirm the presence of the bacterial endosymbionts within the studied freshwater crustacean host species. Phylogenetic analysis supported two main clades, one with all sequences of *Methylophilus* and uncultured bacteria and the second one with *Cardinium*, *Candidatus* Gortzia and *Wolbachia* (Fig. 1). Within these two clades several monophyletic groupings united respective bacterial species (Fig. 1). In total, we revealed the following bacterial endosymbionts in the studied freshwater crustacean hosts: *Wolbachia*, *Methylophilus* and *Candidatus* Gortzia in *B. schaefferi*; *Wolbachia* in *Streptocephalus cafer*; *Cardinium* and *Methylophilus* in *H. incongruens* and *Methylophilus* in *Moina macrocopa*. Moreover, an uncultured bacterium was found in *B. schaefferi*, *B. wolfi*, *M. brachiata* and *Triops cancriformis*. Furthermore, *Undibacterium* belonging to symbiotic microbial communities and endoparasite *Spirobacillus* were identified in *B. schaefferi* (Table 1).

## Discussion

Most aquatic species from temporary waters have a highly specialized life history that allows them to deal with periodic drying (Brendonck, Pinceel & Ortells, 2017). Many of the species produce drought resistant stages that enter a state of dormancy, which can survive for extended periods of time in the absence of water (Brendonck & Meester, 2003). On the individual level, organisms are often time-stressed to complete their life cycle and produce dormant stages before their habitat dries. Under such environmental conditions, shifts in reproductive strategies towards unisexual life cycles seem to be evolutionary beneficial and have been reported by other researchers (Sassaman, 1995; Weeks et al., 2014a; Weeks et al., 2014b; Ford & Weeks, 2018). In terrestrial insects, endosymbiotic bacteria such as *Wolbachia* have been shown to drive such shifts (reviewed in Werren, Baldo & Clark, 2008). However, so far there is a lack of studies on endosymbionts in invertebrates inhabiting temporary waters. Nevertheless, with current high-throughput metagenome analyses, it is possible to study the microbiome and its diversity with a simple probe. This technique is also very useful to identify bacterial symbionts. However, it was shown that analysis of sample composition for individual taxonomic categories of microorganisms, based on homology with databases, very often allows performing identification only to the order level (e.g., Vecchi et al., 2018). If this is the case, bacterial species-specific primers are needed. Primers designed in our study have the advantage that they are very specific to the 16S rRNA of the investigated bacteria and they can be used as a first simple approach to identify potential microbial symbionts associated with freshwater crustaceans. Finally, our simple Sanger sequencing method is cheaper and faster than high-throughput metagenome analyses.

We discovered intracellular *α*-proteobacteria *Wolbachia* in *B. schaefferi* from two Polish astatic ponds and in *S. cafer* from South Africa (Fig. 1, Table 1). *Wolbachia* is a common endosymbiotic bacterium that lives inside host cells (mainly in the ovaries and testes). There are 34,624 sequences of *Wolbachia* in GenBank and that large number illustrates its widespread distribution in various host animals. According to a meta-analysis of Hilgenboecker et al. (2008) as much as 66% of all arthropod species are infected with this endosymbiont. *Wolbachia* plays an important role in evolutionary processes and in shaping the sex structure of the host population (Stouthamer, Breeuwer & Hurst, 1999; Bandi, Trees & Brattig, 2001; Normark, 2003). It has been observed that this bacterium is inherited directly from mother to offspring and evolved various mechanisms for manipulating host reproduction (Funkhouser & Bordenstein, 2013). These include male-killing, feminization, cytoplasmic incompatibility (CI) and induction of parthenogenesis (e.g., Werren, 1997; Zimmer, 2001; Charlat, Hurst & Merçot, 2003). Moreover, its presence may be necessary for embryogenesis and development in some species or may have a beneficial effect on the functioning and life span of the host organism (Brownlie et al., 2009; Nikoh et al., 2014).

For now we can only hypothesize about the nature of the host-microbe relationship in *Wolbachia* and the studied anostracan Crustacea. Interestingly, we did not discover *Wolbachia* in parthenogenetic females of an ostracod *Heterocypris incongruens*. Our results seem to be in line with those of Bruvo et al. (2011) who found no evidence for *Wolbachia* infections in freshwater ostracods. These authors suggest that parasitic infections do not play a role in inducing parthenogenesis in Ostracoda. However, by using our method, we discovered for the first time that *H. incongruens* can serve as a host of bacterial endosymbionts. Therefore, the question arises whether *Cardinium* bacteria could manipulate reproduction of non-marine ostracods. To the best of our knowledge effects of *Cardinium* on host fitness are largely unknown and most reported effects relate to reproductive disorders. So far, screening studies have shown that 6–7% of all arthropod species are infected with *Cardinium* and recent studies have depicted an even higher prevalence of this endosymbiont in chelicerates than in insects (Duron et al., 2008). In addition, Edlund et al. (2012) observed that copepods also served as hosts for endosymbiotic *Cardinium*. Recently, a new group of the *Cardinium* genus infecting biting midges (Diptera: Ceratopogonidae) has been proposed (Nakamura et al., 2009). Endosymbionts of the *Cardinium*-like genus have been also described from the testes and other tissues of the proturan *Acerella muscorum* (Dallai et al., 2011). Until now, 866 sequences of *Cardinium* have been deposited in GenBank.

Four haplotypes of “*Candidatus* Gortzia” were observed in *B. schaefferi* from Poland (Table 1, Fig. 1). Until now, only six sequences of this taxon have been deposited in GenBank which implies that this bacterial endosymbiont is either very rare or difficult to identify. “*Candidatus* Gortzia infectiva”, a member of the order Rickettsiales, belonging to the Holospora-like bacteria (HLB) clade, was first described by Boscaro et al. (2013) as a macronuclear endosymbiont of a free-living ciliate protist *Paramecium jenningsi*. It is capable of horizontal and vertical transmission in the host species and temporary infections of the macronuclei. More recently, “*Candidatus* Gortzia shahrazadis” was also reported as an endosymbiont of *Paramecium multimicronucleatum* by Serra et al. (2016). Since then, no additional reports on these bacteria have been published. According to Serra et al. (2016) it is possible that if exposed to temperature stress, the host reaps the benefit of HLB, since these bacteria are able to enhance heat-shock gene expression of the host. Whether or not this endosymbiont has a functional role in *B. schaefferi* should be the subject of further research.

The methanol-oxidizing bacteria of the genus *Methylophilus* were identified in our study in three phylogenetically distinct species, anostracan *B. schaefferi*, cladoceran *M. macrocopa* as well as an ostracod *H. incongruens* (Table 1, Fig. 1). So far, 745 sequences of *Methylophilus* have been deposited in GenBank but members of this genus were rarely found as endosymbionts of other organisms. Mostly, the presence of *Methylophilus* was reported in urban drinking water distribution systems (Liu et al., 2012). A number of studies have revealed *Methylophilus* strains from the phyllosphere (or above-ground plant structures) of dog rose (*Rosa cinnamomea*), coltsfoot (*Tussilago farfara*) (Gogleva et al., 2010) and thale cress (*Arabidopsis thaliana*) (Reisberg et al., 2012). However, non-singleton phylotypes similar to Methylophilus have been also detected in larvae of four species of Simulium black flies (Tang et al., 2012). Only one report mentions Methylophilus from the cytoplasm and suggests an endosymbiotic relationship of these bacteria with amoeba of the genus Acanthamoeba (Choi et al., 2009). One of our discovered haplotypes (MET 1) was commonly observed in all host species: in B. schaefferi, H. incongruens and M. macrocopa inhibiting the same astatic pond (Poland—Station 3). These results suggest that horizontal transmission of Methylophilus could be possible. However, since this bacterium was also present in cysts of B. schaefferi, also vertical, maternal transmission to next generations through the host cell cytoplasm is likely to occur. Nonetheless, at the current state of knowledge a detailed experimental study is needed to determine the transmission mode of Methylophilus in case of these crustacean taxa.

‘Uncultured bacterium’ records comprised two subclades in our analysis (Fig. 1). In one of these subclades, the sequences of uncultured bacterium were observed in *T. cancriformis* and *M. brachiata*. In the second subclade sequences of uncultured bacterium from *B. schaefferi* and *B. wolfi* were clustered together. It is highly interesting that the most common UCB5 haplotype was observed in both of these anostracan species, one from the site in Poland and one in South Africa (Table 1, Fig. 1). More in-depth study is needed to assess the nature of the interaction between these bacteria and the studied crustacean species. Nevertheless, our results suggest that the bacteria could be endosymbiotic since the phylogenetic relationships among the bacteria are correlated to those among the host taxa. Moreover, in the NCBI database, 258 out of 5,358,795 deposed, Uncultured bacterium” sequences are described as “endosymbiont”.

One sequence was identified as *Spirobacillus* in *B. schaefferi* from Poland (GenBank accession number: MH450240, not shown in Fig. 1). Until now, only 10 sequences of *Spirobacillus* have been deposited in GenBank. *Spirobacillus cienkowskii* is a cladoceran endoparasite (Ebert, 2005). The bacterium infects the hemolymph of its host and changes its color to bright-pink-red through the production of carotenoids (Green, 1959). Although analyzed specimens had a ‘normal’ color, pinkish individuals of *B. schaefferi* were observed in localities where the samples were collected for the present study (Gołdyn & Czyż, pers. comm., 2015). The wide geographical distribution, the ability to infect many different crustacean species and the high virulence indicate that *S. cienkowskii* can play an important role in the ecology and evolution of its hosts (Rodrigues et al., 2008). Infected individuals have a significantly reduced fertility and life span (Duffy et al., 2005). In addition, *S. cienkowskii* may increase the risk of predation due to induction of a more conspicuous color (Rodrigues et al., 2008).

Finally, we identified *Undibacterium* in *B. schaefferi* from Poland (GenBank accession number: mH450134, not shown in Fig. 1). *Undibacterium* is a genus of typically aquatic bacteria, isolated from various sources of freshwater, e.g., drinking water, purified water, waterfalls, freshwater streams, freshwater shrimp culture ponds and also from permafrost soil (Kämpfer et al., 2007; Eder et al., 2011; Liu et al., 2013; Kim et al., 2014; Sheu et al., 2014a; Sheu et al., 2014b; Du et al., 2015; Nguyen, Lee & Kim, 2015; Chen et al., 2017). In addition, it was isolated from shrimp intestines (Rungrassamee et al., 2014) and from zebrafish *Danio rerio* (Kämpfer et al., 2016). The genus *Undibacterium* has been described in host-associated symbiotic microbial communities (Jani & Briggs, 2014) and 132 sequences of *Undibacterium* have been deposited in GenBank.

## Conclusion

Until now, sequencing of unknown bacterial endosymbionts was very challenging and species-specific primers were necessary to amplify 16S rRNA sequences of each specific bacterial endosymbiont. Nevertheless, primer specificity not always allows for detection of bacterial endosymbionts from a sample containing a large variety of microorganisms. Under such conditions interfering PCR products confound the results. Our newly designed non-degenerate primers allow amplification of the 16S rRNA region of different bacterial species with an efficiency of 100%. By applying the method, we were able to deliver the first evidence of the presence of various bacterial endosymbionts in several freshwater crustacean host species. Therefore, we propose to use the newly designed WOLBSL and WOLBSR primers at the first stage of endosymbiont bacterial identification. Subsequently, commonly used primers for amplifying specific 16S rRNA regions of given bacteria can be used.

The newly designed primers amplify only prokaryotic 16S rRNA, since we obtained only sequences of bacteria and none that could be ascribed to any of the host species. Main advantages of our primer approach include a high specificity to bacterial DNA and reduced expenses due to a high sequencing yield. Furthermore, the primers are generic with regard to the host on which they are applicable. Overall, we believe that our fast and simple identification method of bacterial endosymbionts will facilitate studies on interactions and co-speciation between bacteria and invertebrates inhabiting temporary ponds.

##  Supplemental Information

10.7717/peerj.6039/supp-1 Supplemental Information 1 The 16S rRNA sequences of Candidatus Gortzia endosymbiont of *Branchipus schaefferi*

10.7717/peerj.6039/supp-2 Supplemental Information 2 The 16S rRNA sequences of Cardinium endosymbiont of *Heterocypris incongruens*

10.7717/peerj.6039/supp-3 Supplemental Information 3 The 16S rRNA sequences of Methylophilus amplified from crustacea species

10.7717/peerj.6039/supp-4 Supplemental Information 4 The 16S rRNA sequence of Spirobacillus amplified from *Branchipus schaefferi*

10.7717/peerj.6039/supp-5 Supplemental Information 5 The 16S rRNA sequences of Uncultured bacterium of Crustacea

10.7717/peerj.6039/supp-6 Supplemental Information 6 The 16S rRNA sequence of Undibacterium amplified from *Branchipus schaefferi*

10.7717/peerj.6039/supp-7 Supplemental Information 7 The 16S rRNA sequence of Wolbachia endosymbiont of *Streptocephalus cafer*

10.7717/peerj.6039/supp-8 Supplemental Information 8 The 16S rRNA sequence of Wolbachia endosymbiont of *Branchipus schaefferi*

10.7717/peerj.6039/supp-9 Supplemental Information 9 The 16S rRNA sequences of Wolbachia endosymbiont of *Branchipus schaefferi*

## Additional Information and Declarations

### DNA Deposition

The following information was supplied regarding the deposition of DNA sequences: The bacterial 16S rRNA sequences described here are accessible via GenBank accession numbers: MH376280 to MH376282, MH447357, MH447361 to MH447365, MH447385 to MH447392, MH447689, to MH447702, MH450134, MH450232, to MH450236, MH450240, MH454491.

### Data Availability

The following information was supplied regarding data availability: The raw measurements are provided in the Supplemental Files.
